# Cardiovascular magnetic resonance in familial amyloidosis

**DOI:** 10.1186/1532-429X-18-S1-P266

**Published:** 2016-01-27

**Authors:** Maria A Espinoza Barillas

**Affiliations:** Radiology, Instituto Nacional De Ciencias Medicas Y Nutricion Salvador Zubiran, Mexico DF, Nicaragua

## Background

Endomyocardial biopsy is considered the gold standard tool, but it is an invasive method, with low sensitivity and specificity, the echocardiographic diagnosis is difficult. MRI has recently emerged as a noninvasive technique that provides morphological, functional and tissue characterization information in evaluating this condition.

## Methods

Our objective was to identify morphological and functional findings of familial amyloidosis in Cardiovascular Magnetic Resonance and compare this findings between asymptomatic patients and those with cardiovascular symptoms. Cardiovascular Magnetic Resonance was performed in 19 patients with familiar amyloidosis, 9 of them with cardiovascular symptoms, the remaining 10 were asymptomatic. The diagnosis of amyloidosis was previously done by periumbilical fat biopsy and genetic studies.

## Results

The LVEF was preserved in 74% of the cases but 90% had alterations in global contractility. The atrioventricular valves were thickened in all patients. The right ventricle and atria were affected in 26% and 47% respectively. The most frequent pattern of enhancement in late gadolinium phase was suboptimal myocardial nulling (36%), followed by global transmural heterogeneous pattern.

## Conclusions

Suboptimal myocardial nulling was the most common pattern of late gadolinium enhancement found in this study, its present even in the absence of morphological changes.

There was no statistically significant differences in the proportions of the structural and functional parameters in patients with cardiovascular symptoms and those asymptomatic.

**Figure 1:**
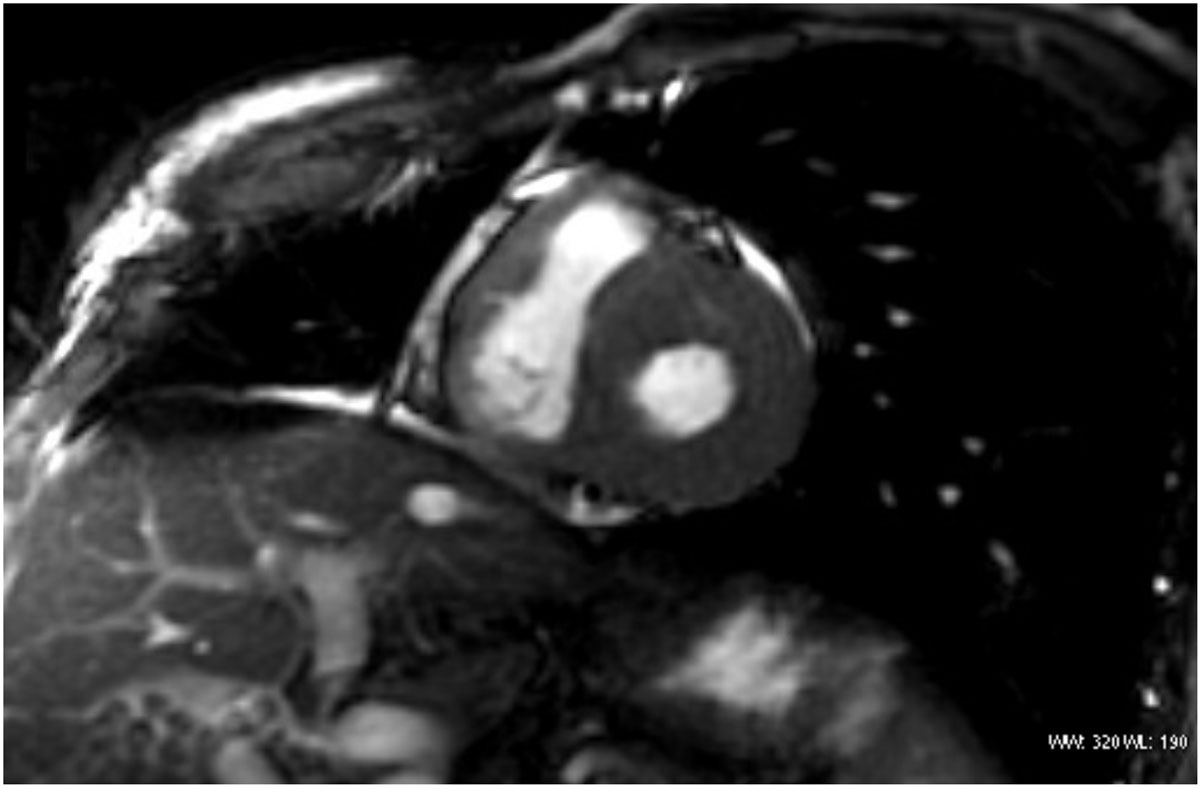
**Short axis in Steady state free precession**. Note walls thickening of both ventricles.

**Figure 2:**
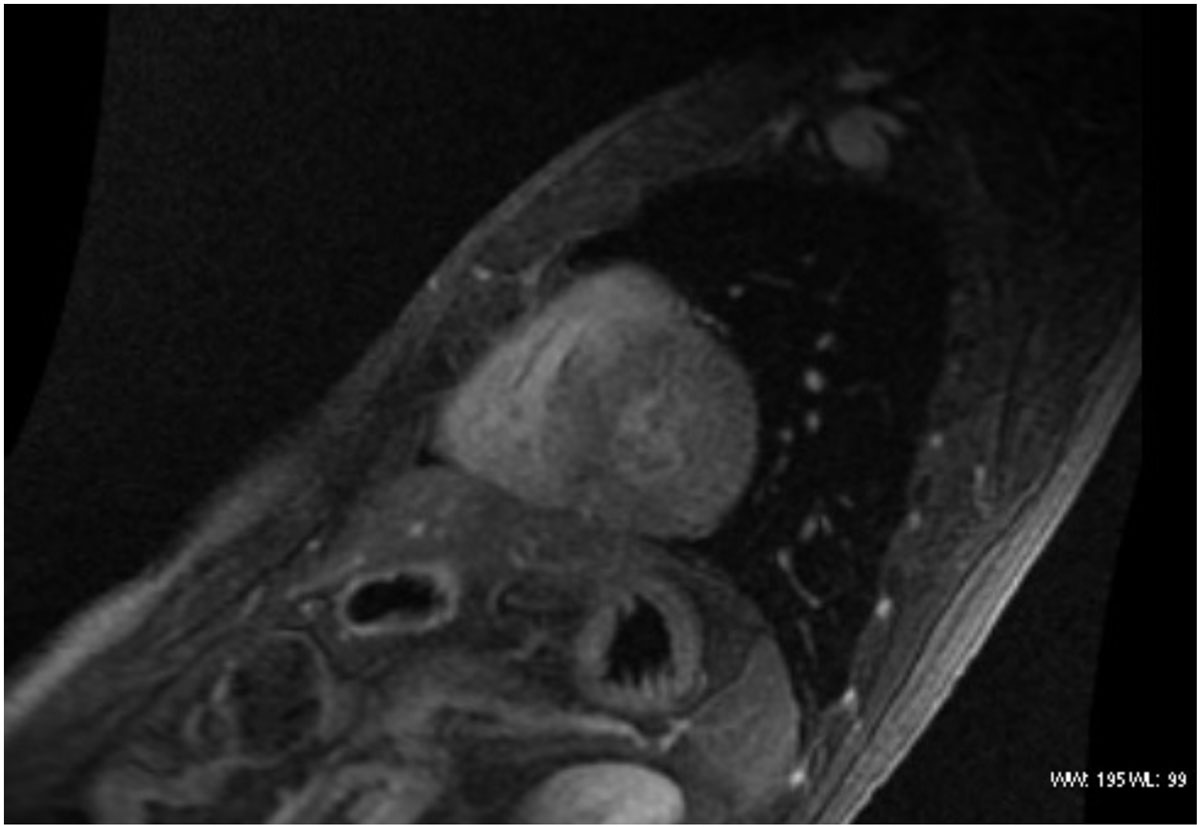
**Short axis view**. Late gadolinium enhancement with suboptimal myocardial nullification pattern.

